# Randomized controlled trial comparing four strategies for delivering e-curriculum to health care professionals [ISRCTN88148532]

**DOI:** 10.1186/1472-6920-6-2

**Published:** 2006-01-11

**Authors:** Kathi J Kemper, Paula Gardiner, Jessica Gobble, Ananda Mitra, Charles Woods

**Affiliations:** 1 Pediatrics and Public Health Sciences, Wake Forest University School of Medicine, Winston-Salem, NC, 27157, USA; 2 Division for Research and Education in Complementary and Integrative Medical Therapies, Harvard Medical School, Osher Institute, Boston, MA, 02115, USA; 3 Northwest Area Health Education Center, Wake Forest University School of Medicine, Winston-Salem, NC, 27157, USA; 4 Department of Communications at Wake Forest University, Winston-Salem, NC, 27157, USA

## Abstract

**Background:**

Internet education is increasingly provided to health professionals, but little is known about the most effective strategies for delivering the content. The purpose of this study is to compare four strategies for delivering an Internet-based (e-) curriculum on clinicians' knowledge (K), confidence (CONF), and communication (COMM) about herbs and other dietary supplements (HDS).

**Methods:**

This national randomized 2 × 2 factorial trial included physicians, pharmacists, nurses, nutritionists and trainees in these fields. Participants were randomly assigned to one of four curriculum delivery strategies for 40 brief modules about HDS: a) delivering four (4) modules weekly over ten (10) weeks by email (drip-push); b) modules accessible on web site with 4 reminders weekly for 10 weeks (drip-pull); c) 40 modules delivered within 4 days by email (bolus-push); and d) 40 modules available on the Internet with one email informing participants of availability (bolus-pull).

**Results:**

Of the 1,267 enrollees, 25% were male; the average age was 40 years. The completion rate was 62%, without significant differences between delivery groups. There were statistically significant improvements in K, CONF and COMM scores after the course (P<0.001 for all), although the difference in COMM was small. There were no significant differences in any of the three outcomes by delivery strategy, but outcomes were better for those who paid for continuing education credit.

**Conclusion:**

All delivery strategies tested similarly improved K, CONF, COMM scores about HDS. Educators can use the strategy that is most convenient without diminishing effectiveness. Additional curricula may be necessary to make substantial changes in clinicians' communication practices.

## Background

Herbs and dietary supplements (HDS) are the most commonly used complementary or alternative medical (CAM) commodities in the US [1]. Substantial numbers of people report using HDS. However, fewer than half of patients who use HDS typically discuss it with their clinician [2], in part because clinicians do not consistently inquire and in part because patients do not perceive health care professionals as particularly knowledgeable about HDS [3].

Clinicians have been urged to initiate routine discussions about HDS with patients, but few do so routinely [4]. Clinicians have expressed strong interest in training about CAM [5,6]. For example, in a national survey of pediatricians, over 80% reported wanting additional training about HDS [7]. To begin to address this educational need, we conducted a randomized cross-over pilot study of 537 health professionals in 2000 to determine whether an Internet-based or e-curriculum could improve knowledge (K), confidence (CONF) and communication (COMM) practices about HDS. The curriculum consisted of a moderated Listserv and 20 cased-based modules with links to evidence-based information on the Internet. Two modules were delivered to participants by email each week for ten weeks ("drip-push" method of delivery). The curriculum was associated with significant and sustained improvements in K, CONF and COMM practices [8].

Although the e-curriculum was effective, it was challenging to deliver. Problems included changes in participants' email addresses, "firewalls" at various institutions that blocked email delivery, and "full" mailboxes that could not accept incoming messages, leading to incomplete delivery.

Delivering curriculum, as we did in the pilot study, in small amounts over a period of time ("drip delivery") is appealing educationally, but it is not clear that the theoretical advantage of easier absorption with this approach outweigh its logistical challenges over a "bolus" (delivery over a short time). Furthermore, although delivering the curriculum by email ("pushing" the curriculum) has theoretical advantages over posting content on a web site ("pulling" the learner to the curriculum) since "pushing" decreases the number of "clicks" the participant must use to access the content, it is not clear that these advantages outweigh the challenges of a "push" delivery.

Therefore, we performed a randomized controlled trial (RCT) using a 2 × 2 factorial design to compare the impact of "drip" versus. "bolus" (small amounts of material delivered over time vs. a large amount in a short time) and "push" versus "pull" (delivery by email vs. availability at project web site) on clinicians' and trainees' K, CONF and COMM practices regarding HDS.

## Methods

This was a randomized, controlled trial comparing four different strategies for delivering an e-curriculum about HDS to health care professionals. Subjects were eligible if they were physicians, physician assistants, nurses, pharmacists, nutritionists or dietitians, or trainees in one of these fields.

### Recruitment

In summer, 2004 the Northwest Area Health Education Center of North Carolina (NW AHEC) sent emails to approximately 27,000 individuals in its state-wide continuing education (CE) database announcing the availability of the curriculum. Emails were also sent to department chairs for medicine, nursing, pharmacy and nutrition at North Carolina (NC) health professions schools. The Principal Investigator (KK) also emailed invitations to colleagues, personal contacts and professional list-serve groups. Overall, we sent approximately 29,000 email announcements. "Viral" marketing (i.e., forwarding of the original emails) was not formally monitored. Approximately 500 flyers were also distributed at various continuing education (CE) activities of NW AHEC.

In similar fashion, in spring, 2005, we sent email announcements to the same groups as for fall, 2004. In addition, emails were sent to the South Carolina AHEC and to faculty at South Carolina schools of nursing, pharmacy, nutrition, and medicine that were listed on the Internet. Emails were also sent to the Wake Forest University School of Medicine (WFUSM) Alumni Association and listservs for the Ambulatory Pediatric Association and the Society of Teachers of Family Medicine. We also sent a notice to the listserv for the 27 members of the Consortium of Academic Health Centers for Integrative Medicine (Consortium); two of these Consortium-affiliated medical schools promoted the program to their students by email. We sent approximately 30,000 email announcements for spring. Brochures were mailed to 19,000 persons on the North Carolina AHEC health professions database and the WFUSM Office of Continuing Medical Education (CME) database who did not have email addresses listed.

Participants registered on-line through the NW AHEC website. Following registration, participants completed the baseline survey on-line. After completing the baseline survey, the 1267 enrollees were randomized by a computer-generated randomization program (Microsoft Excel^®^) to one of four strategies for receiving the 40 modules of the curriculum: Drip-Push, Drip-Pull, Bolus-Push and Bolus-Pull, described above.

### Curriculum

The curriculum consisted of two elements: a moderated listserv and 40 brief modules. All participants were enrolled in the listerv, a moderated electronic discussion group, in which participants were encouraged to post clinical questions and to respond other participants' questions. Questions and responses were screened to avoid posting advertisements, personal messages, and junk mail. Postings were made in batches to the Listserv once or twice weekly, and all postings were available on the project web site. Participants could withdraw at any time by sending an email request to the project coordinator; only 7/1267 (<1%) did so.

The second curricular element was a set of 40 case-based, self-instructional modules; each module contained a one to two sentence case, a multiple-choice question and the answer. The question/answer format was designed to mimic a clinical situation to promote learning; it was not used to score outcomes. The 40 modules were divided into 10 content areas such as aging, cardiovascular disease, and women's health. Modules contained three to 19 links to evidence-based Internet resources about HDS (see Appendix 1 for example of a module). Most links were to original research articles on PubMed; other links included government sites such as the National Institutes of Health (NIH) Office of Dietary Supplements, the NIH National Center for Complementary and Alternative Medicine, and the National Cancer Institute; non-profit health groups such as the American Cancer Society; and academic sites such as Memorial Sloan-Kettering Cancer Institute. The modules were developed originally by physicians, pharmacists, nurses and dietitians; the modules underwent extensive pilot testing and revision prior to use for this study [8]. Each module required an average of less than 12 minutes to complete, including accessing links. The text in the modules themselves provided the answers to questions on the baseline and follow-up surveys.

### Baseline and outcome measures

The survey questions for this project were based on those used in our pilot study [9]. In addition to demographic data, we asked about participants' profession, whether they were students or trainees, whether they had seen any patients in the 30 days prior to the survey, and about their own use of HDS. At baseline, we also asked them to rate their computer knowledge on a scale from 1 to 7, with 1 = Novice and 7 = Trainer/Specialist.

*Knowledge scores *were generated as percent of the knowledge questions answered correctly. Questions included items about the use, effectiveness and safety of commonly used HDS such as green tea, St. Johns wort, ephedra, folate, fish oil and glucosamine. Scores could range from 0 to 100% correct.

A *confidence scale *score was derived from responses to 19 Likert-type questions (strongly disagree, disagree, neutral/not sure, agree, strongly agree) such as "I feel confident responding to patients' questions about HDS." (See Appendix 2.) Each item was scored 1 (strongly disagree) to 5 (strongly agree), with a minimum score of 19 and maximum of 95. The Cronbach alpha reliability statistic was 0.96 for the confidence scale for both baseline and outcome assessments.

An 11-item *communications practices scale *was used for those respondents who had seen patients within the past 30 days. (See Appendix 2.) Nine items asked for responses in 10% increments from 0 to 100% (e.g., "In the past 30 days, in what percentage of your clinical encounters have you discussed with a patient or family the use of HDS?"). Two questions were in yes-no format: whether in the past 30 days, respondents had: 1) cautioned any patient about potential hazards of HDS; or 2) discussed a question about HDS with any colleagues. The first nine items were scored as a proportion corresponding to the percentage chosen (0.0 to 1.0); the two yes-no items were scored as 0.5 for yes and 0 for no. The possible range of scale scores was 0 to 10. For the COMM practices scale, the Cronbach alpha reliability statistic was 0.84 for baseline and 0.92 for the outcome assessments.

For purposes of this analysis, course completion was defined as answering all of the knowledge or confidence questions on the outcome survey. The outcome survey also included several optional questions to provide qualitative feedback about the curriculum.

Continuing Education (CE) credit was available for clinicians who completed both the baseline and outcome surveys at a nominal fee ($35); the course was free for trainees and others not seeking CE credit (auditors).

### Analysis

Descriptive statistics were generated using means and standard deviations for normally distributed data and medians for non-normally distributed data. Two-way comparisons were tested by Chi-square for nominal and categorical data, using analysis of variance (ANOVA) for normally distributed data and non-parametric tests such as Mann-Whitney U tests and Kruskal-Wallis tests for non-normally distributed variables. For repeated measures outcomes, paired samples t-tests or Wilcoxon signed rank tests were used, depending on data characteristics. Spearman rank correlation was used to assess association between ordinal and continuous variables. Backward conditional multivariable logistic regression was performed to assess the relative importance of individual factors affecting outcomes. P value < 0.05 was required for retention in the model. Analyses were performed using SPSS 13.0 (SPSS Inc., Chicago, IL).

This study was approved as "exempt" as an educational research project by the Wake Forest University School of Medicine Institutional Review Board.

## Results

Of the 1267 enrollees, 780 (62%) completed the outcome survey. On average, enrollees were 40 years old; 25% were male (Table 1). There were no significant differences at baseline among the four different curriculum delivery groups by age; gender; profession; race; ethnicity; geographic location; personal use of HDS; self-appraisal of computer knowledge; or baseline scores on K, CONF or COMM practices, indicating that randomization resulted in equivalent groups (Table 1).

### Use of resources

For the listserv, there 44 postings in the Fall and 112 for the Spring. No participant attempted to post junk mail or advertisements.

Due to the technical nature of the links for each module, we could ascertain the number of "hits" to each link, comparing "push" to "pull", but not the "drip" to "bolus" groups. Altogether, there were 38,713 hits to 335 links. The average number of hits per link was significantly higher for "push" than for "pull" users (63.1 versus 52.4 hits per link, P < 0.01). Hit rates fell over the 10-week curriculum, with an average of over 90 hits per link in week 1, falling to 43 hits per link in week 9. There was no significant relationship between the number of links provided in a module and the hit rate for those links. The most frequently hit links were Internet sites that provided reviews, guidelines, summaries and patient handouts rather than links to original research.

### Course completion

Completion rates did not differ by delivery strategy in the bivariate analysis (Table 1). However, course completers compared with non-completers had a statistically significantly higher average age, used a higher number of HDS personally, had lower scores on self-appraised computer knowledge, had higher baseline scores on K and CONF and were more likely to seek CE credit for the course (P<0.05 for all comparisons, Table 2). Also, course completers were more likely to be female, to have seen a patient in the 30 days prior to enrollment, and less likely to be physicians or students than were non-completers (P<0.05 for all comparisons). Since many of these factors may be inter-related, we performed multivariable logistic regression to determine which modifiable factors were most strongly associated with course completion. In the multiple logistic regression analysis, participants who paid a fee for CE were 3.3 times more likely to complete the course than auditors (odds ratio [OR] 3.27; 95% confidence interval [CI]: 2.39, 4.47, P<0.001). Also, in the multiple regression analysis, delivery strategy emerged as a significant predictor of completion, with those receiving "drip" having a 30% greater likelihood of completing than those who received "bolus" delivery (OR 1.31; 95% CI: 1.02, 1.68, P = 0.03).

### Primary outcomes

Course completers had significant improvements in knowledge from 67% correct at baseline to 89% at outcome, P<0.001 by paired t-test, without significant differences by delivery strategy (Table 3). In bivariate analyses, other factors were significantly associated with greater improvements in K scores (Table 4). These included: having paid the CE fee; age ≤ 30 years old; being a nurse, nutritionist or student (versus being a physician or pharmacist); using fewer HDS at baseline; and reporting less computer expertise (P<0.05 for all comparisons). In the regression analysis, the most potent factor affecting improvement in K score was paying the CE fee (P < 0.001); however, professional group, age and personal use of HDS were retained in the model with P < .05.

Course completers also had significant and substantial improvements in CONF (improvement from 53.8 to 64.3 on the CONF scale, *P *< 0.001 by paired t-test), with similar improvements for the four different delivery strategies (Table 3). Other variables significantly enhanced improvements in CONF (Table 4): being a trainee; not having seen patients in the 30 days prior to enrollment; having paid for CE; age ≤ 30 or > 50 years old; and being a nurse or student (versus other health professionals). In the multivariable analysis, the strongest predictor of improvements in CONF was paying the CE fee (*P *< 0.0001); other factors that remained significant included professional group (nurse) and being a trainee.

Finally, course completers had small, but statistically significant improvements in COMM (improvement from 1.67 to 2.0 on the 10-point scale, *P *< 0.01). These improvements also did not differ by assignment to different curriculum delivery strategies (Table 3). Because improvements in COMM were unaffected by any other variables in bivariate analyses, no multivariate analyses were performed for this outcome.

### Qualitative assessment

Qualitative comments were overwhelmingly positive about the course content, the modules' case-based question/answer format, course organization, links, and the Listserv. Participants frequently complimented its convenience, low cost, breadth and links to reliable sources of information. One participant noted that she stopped using the links after the first few modules, because she had learned to trust that the links supported the text.

Constructive feedback was almost entirely about the volume and delivery method for the course. For example, "It was just too much for me to keep up." There were frequent suggestions to "decrease the number of modules and links", particularly from those who received the bolus delivery. On the other hand, some in the "drip" group said they would have preferred "receiving all the modules at once up front" so they could take their time to review them. Some who received the curriculum by email occasionally stated that a preference for an online program because of difficulty receiving emails. On the other hand, some of those who received the "pull" delivery stated that they would have preferred delivery by email. There were many requests for additional similar courses, ongoing updates, printed reference materials, a searchable subject index on the project website, patient handouts, clinical tools for recording patients' use of HDS, treatment plans or guidelines that include HDS, and providing the course and patient resources in Spanish.

## Discussion

To our knowledge, this is the first RCT comparing the impact of four different strategies for delivering an e-curriculum on HDS for practicing and in-training clinicians. There was a slightly higher completion rate for those receiving the "drip" compared with the "bolus" delivery method, and there were more "hits" to Internet links for the "push" than the "pull" strategy, but these differences did not appear to affect the primary outcomes.

In fact, the delivery strategy appears to have far less impact on key outcomes than whether or not participants paid for CE credit. Those who paid for credit were three times more likely than auditors to complete the outcome surveys. This finding suggests that when trying to minimize barriers to participation (by making enrollment free or low cost), participation and course completion may be enhanced by requiring at least a nominal fee.

Improvements were greater among those participants with lower scores at baseline (e.g., such as students). Unexpectedly, participants who did not consider themselves experts in using computers did just as well as those who rated their computer skills more highly, suggesting that Internet-based curricula can be effective even for those with only modest computer skills. Most participants preferred to have fewer modules per course and have more courses available over a longer period of time. Ancillary materials, resources and content indexing would enhance the utility of the curriculum as an ongoing reference. These data and qualitative feedback provide key information for planning future courses.

The most urgent and unmet educational needs appear to be for improving communication skills [10]. Self reported rates may substantially over-estimate the actual rate of desirable behaviors [11]. Participants' improvements in COMM in this study were statistically significant, but small; other types, amounts, intensities or duration of curricula may be required to improve clinicians' communication behaviors. Clinicians' behavior can be improved with extensive training and personal experience 54% [12,13], but it is not clear whether e-curriculum can substantially improve communication practices; further research on this question is urgently needed.

The strengths of our study are its large and multidisciplinary sample; the randomized controlled, factorial design; the use of a curriculum already established as effective in a large pilot study; the use of outcome measures with excellent internal validity as measured by Cronbach's alpha; objective data about actual use of some parts of the curriculum; and the inclusion of qualitative as well as quantitative outcomes. We replicated our earlier findings that an e-curriculum could significantly improve K & CONF, and we extended these findings by showing the equivalent outcomes from four different delivery strategies.

However, this study has several limitations. First, the sample was highly self-selected to those who were interested in learning more about HDS, which limits the generalizability of our findings to elective (versus required) courses and possibly to courses about CAM topics rather than more traditional CME topics. The sample was electronically literate, having completed enrollment, the baseline survey, the curriculum and the outcome survey entirely on the Internet. Like most educational research, this study relied on self-report rather than observation of clinical practice; future studies should directly measure impact on behavior as well as self-report. Future studies also will need to address the effectiveness and costs of various curricular components such as the links and the listserv. This study could also not address the relative impact of different delivery strategies for courses of different lengths or intensities; there may be different effects for courses that are substantially longer or shorter than this one.

## Conclusion

Despite these limitations, results from this study have important implications for professional education and future research. Educators who wish to use the Internet can be confident that improvements are not heavily dependent on the curriculum delivery strategy, at least among the four strategies tested in this study. In fact, the delivery method that is easiest for instructors (bolus-push), may present the fewest barriers for completion (such as full mailboxes and institutional firewalls), particularly for short, introductory courses. At any rate, the delivery strategy that is easiest does not appear to result in substantially worse outcomes. Additional interventions are needed to improve clinicians' communication behavior. This study represents an important first step – we have demonstrated that Internet education can substantially improve clinicians' knowledge and confidence regardless of the delivery strategy; next, innovative, cost-effective strategies to improve behavior and clinical outcomes need to be developed and evaluated.

## Abbreviations

ANOVA = Analysis of Variance

CAM = Complementary and Alternative Medicine

CE = Continuing Education

CME = Continuing Medical Education

COMM = communication practices

CONF = confidence

HDS = herbs and dietary supplements

K = Knowledge

NC = North Carolina

NIH = National Institutes of Health

NW AHEC = Northwest Area Health Education Center of North Carolina

RCT = Randomized Controlled Trial

## Appendix 1: Example of one of the Modules: Nausea module

*CASE*: A 33 year old woman is suffering from nausea associated with her pregnancy. She asks if there are any safe, natural remedies to ease her nausea.

*QUESTION*: Which ONE of the following is FALSE?

A) Several randomized controlled trials support the use of ginger in the treatment of nausea.

B) Ginger causes neural tube defects and should not be used during pregnancy.

C) Some pregnant women find that Vitamin B6 helps ease nausea symptoms.

D) Peppermint's effectiveness as an oral agent in treating nausea associated with pregnancy has not undergone rigorous randomized controlled trials.

*ANSWER*: **B is false. A, C and D are true.**

Over 70% of pregnant women experience nausea. Many of these women use home remedies or complementary therapies. The top three complementary therapies used for nausea associated with pregnancy are: ginger, vitamin B6 and acupressure bands.

*Ginger *is a safe spice that is widely used in cooking. It does not cause birth defects or have any other serious toxicity; see safety data from randomized controlled trial, published in *Am J Obstet Gynecol *in 2003. See the Longwood Herbal Task Force review of ginger. The dose of ginger most often used in these studies is 500 mg TID – QID.

A meta-analysis concluded that *Vitamin B6 *(pyridoxine) offered significant benefits in reducing nausea associated with pregnancy. A randomized, controlled trial involving 291 pregnant women showed that vitamin B6 and ginger were similarly effective in reducing nausea symptoms; see study in 2004 *Obstet Gyncol*. A similar study was reported among Thai women – both ginger and vitamin B6 offered significant benefits, had few side effects and were similar to one another. The dose of Vitamin B6 most often used is 10 mg – 25 mg TID.

Some women also use peppermint to help reduce nausea symptoms during pregnancy. One trial showed that peppermint significantly improved postoperative nausea. However, more recent a randomized controlled trial concluded that peppermint *aromatherapy *was no more effective than placebo in alleviating postoperative nausea. Studies have not been done in the setting of nausea associated with pregnancy, but it is generally recognized as safe. See the Longwood Herbal Task Force reviews on peppermint.

(underlined text indicates link to internet site with evidence-based information)

## APPENDIX 2 (CONFIDENCE AND COMMUNICATION PRACTICES SCALES)

Confidence Scale

For each item, respondents are asked to indicate how they feel about each statement on a 5-point Likert scale including Strongly Disagree, Disagree, Neutral, Agree and Strongly Agree.

1. I feel confident responding to *patients' questions *about H/DS.

2. I feel confident *initiating discussions *with patients about H/DS.

3. I know how to ask about which *brands and doses *patients are using of H/DS.

4. I can *warn patients *about *side effects *of commonly used H/DS.

5. I can *warn *patients about *interactions *between commonly used H/DS and medications

6. I can *provide *evidence-based information about H/DS to patients.

7. I can refer patients where to find information about the *quality *of different brands of H/DS.

8. I can tell my patients about the appropriate dose and duration to use H/DS.

9. I know where to *refer *patients for more information about H/DS

10. I know where *I *can turn for reliable information about H/DS.

11. I can readily *record *information about patients' use of H/DS in the patient record.

12. I feel confident talking with *colleagues *about H/DS.

13. I know more about H/DS than many health care providers.

14. I know where and how to *report adverse effects *related to H/DS.

15. I could give a short lecture or demonstration to my colleagues about H/DS.

16. If a reporter or magazine writer called, I could answer questions about H/DS.

17. I can write a letter to the editor or a short review article about H/DS.

18. I can teach a high school science class about H/DS.

19. I can give a lecture about H/DS for students in my profession.

## Communication practices scale (answered only by participants who reported having seen a patient in the 30 days prior to survey)

For each of the following questions, respondents were asked to estimate to the nearest 10% (from 0% to 100%). Potential responses were provided in a drop-down box on the web-based data entry screen.

1. In the past 30 days, in what percentage of your clinical encounters have you *discussed with a patient or family *the use of herbs or other dietary supplements?

2. In what percentage of these encounters did YOU initiate the discussion about herbs and supplements?

3. In the past 30 days, in what percentage of your patient encounters did you ask about the *brand name or manufacturer *of the herbs and dietary supplements used by your patients?

4. In the past 30 days, in what percentage of your patient encounters did you ask about the *dose *(amount and frequency) of herbs and dietary supplements?

5. In the past 30 days, in what percentage of your patient encounters did you ask about the *side effects *of herbs and dietary supplements used by your patients?

6. In the past 30 days, in what percentage of your patient encounters did you *provide patient handouts *or refer patients/families to specific books, articles or web sites for additional information about herbs and dietary supplements?

7. In the past 30 days, in what percentage of your patient encounters did you record the patients' use/non-use of herbs and dietary supplements in the *patient record*?

8. In the past 30 days, in what percentage of your patient encounters in what percent of patient *records *did you note an *adverse event *from an herb or supplement?

9. In the past 30 days, in what percentage of your patient encounters did you *note an interaction *between the herb or supplement and a medication?

The last two items were Yes/No responses, scored as No = 0 and Yes = 0.5

10. In the past 30 days, have you cautioned any patient about the potential hazards associated with the use of any herbal products (other than tobacco)?

11. In the past 30 days, have you *discussed with a colleague *a clinical question related to the use of herbs or dietary supplements?

## Competing interests

The authors declare that in the past five years, we have not received reimbursements, fees, funding or salary from an organization that may in any way gain or lose financially from the publication of this manuscript, either now or in the future. We all work for non-profit academic institutions. We do not hold stocks or shares in any organization that may in any way gain or lose financially from the publication of this manuscript, either now or in the future. We do not hold and are not currently applying for any patents related to the content of this manuscript. We have not received reimbursements, fees, funding or salary from an organization that holds or has applied for patents relating to the content of the manuscript. We do not have other financial competing interests. Nor do we have any non-financial competing interests to declare in relation to this manuscript.

## Authors' contributions

*KK *was responsible initiating, creating and designing this project, content development, overseeing the entire project and drafting, revising and submitting this manuscript.

*PG *was responsible for assisting with the content of the curriculum as an expert on herbal medicine, and for assisting in revising the manuscript.

*JG *was the project manager for this project, responsible for assisting with web site design, marketing, data collection, editing content, assisting in management of the Listserv, assisting with revising and editing the manuscript and contributing the writing for the methods section.

*AM *was responsible for the portions of the survey dealing with computer expertise, contributing to study design for the push versus pull element, revising and editing the manuscript.

*CW *was responsible for assisting with study design, statistical analysis, and writing the portions of the manuscript related to data analysis and interpretation.

All authors read and approved the final manuscript.

## Pre-publication history

The pre-publication history for this paper can be accessed here:



## Figures and Tables

**Table 1:** Baseline characteristics of the study subjects by randomized groups.

**Characteristic**	**Total**	**Randomized Group**				**P Value**
		
		**Push + drip**	**Push + bolus**	**Pull + drip**	**Pull + bolus**	
**N**	1267	318	318	318	313	
**Age (mean ± SD)**	40.3 ± 12.9	41.2 ± 12.8	40.1 ± 12.3	40.2 ± 13.3	39.9 ± 13.1	.58†
**Gender (% male)**	25.3%	28.9%	21.1%	26.4%	24.9%	.14*
**Race, N (%)**						.09*
African Am	57 (4.5)	9 (2.8)	11 (3.5)	18 (5.7)	19 (6.1)	
Asian/P.I.	96 (7.6)	18 (5.7)	30 (9.4)	25 (7.9)	21 (7.3)	
Caucasian	1052 (83.0)	276 (86.8)	261 (82.1)	257 (80.8)	257 (82.1)	
Native American	3 (0.2)	0	3 (0.9)	0	0	
Declined	60 (4.7)	15 (4.7)	13 (4.1)	18 (5.7)	14 (4.5)	
**Latino origin?**						
Yes	41 (3.2)	13 (4.1)	7 (2.2)	11 (3.5)	10 (3.2)	.85*
**Profession**						.77*
MD	374 (29.5)	99 (31.1)	100 (31.4)	87 (27.4)	87 (27.8)	
PHARM	58 (4.6)	15 (4.7)	17 (5.3)	11 (3.5)	15 (4.8)	
RD	150 (11.8)	31 (9.7)	41 (12.9)	35 (11.0)	43 (13.7)	
RN	296 (23.3)	78 (24.5)	65 (20.4)	82 (25.8)	71 (22.7)	
Student	390 (30.8)	95 (29.9)	95 (29.9)	103 (32.4)	97 (31.0)	
						
**Residence (% NC)**	47.2%	45.9%	48.4%	47.8%	46.6%	.92*
**Faculty/Practice (vs in training)**	727 (57.3)	184 (57.9)	184 (57.7)	185 (58.2)	174 (55.6)	.91*
**Has seen patients in past 30 days**	852 (67.2)	210 (66.0)	228 (71.7)	212 (66.7)	202 (64.5)	.24*
**Any Herb Use**	1079 (85.1)	270 (84.9)	281 (88.4)	263 (82.7)	264 (84.3)	.24*
**HDS use (mean ± SD)**	5.5 ± 6.3	5.6 ± 6.2	5.5 ± 6.0	5.5 ± 6.8	5.5 ± 6.3	.86‡
**Self rank on computer knowledge^**1**^**	4.37 ± 1.15	4.32 ± 1.16	4.46 ± 1.33	4.37 ± 1.12	4.35 ± 1.19	.49†
**%Knowledge questions correct**	65.8 ± 10.7	65.6 ± 10.7	66.7 ± 10.2	64.8 ± 10.7	66.1 ± 10.8	.12†
**Confidence scale score (range: 19,95)**	52.5 ± 18.2	52.4 ± 17.9	52.8 ± 17.8	52.5 ± 18.2	52.3 ± 17.8	.99†
**Practice Scale (range 0,10)**	2.2 ± 1.9	2.1 ± 1.8	2.4 ± 2.0	2.1 ± 2.0	2.2 ± 1.9	.24‡
**Paid CE Fee**	587 (46.3)	149 (46.9)	153 (48.1)	148 (46.5)	137 (43.8)	.74*
**Completed outcome**	780 (61.6)	195 (61.3)	206 (64.8)	177 (56.5)	202 (63.5)	.16 *

* Determined by Chi square method.

† Determined by One-way ANOVA.

‡ Determined by Kruskal Wallis test.

^1 ^On this self-report scale, 1 = Novice and 7 = Trainer/Specialist.

**Table 2:** Associations of subject characteristics with course completion.

**Characteristic**	**Non-Completion**	**Completion**	**P Value**
N	487	780	
**Age **(mean ± SD)	37.4 ± 12.8	42.2 ± 12.6	<.001*
**Gender**			
Male	143 (44.5)	178 (55.5)	.011†
Female	344 (36.4)	602 (63.6)	
**Profession**			<.001†,‡
MD	140 (37.5)	233 (62.5)	
RD	38 (25.3)	112 (74.7)	
RN	86 (29.1)	210 (70.9)	
Pharm	14 (24.1)	44 (75.9)	
Student	209 (53.6)	181 (46.4)	
**Delivery Strategy**			
Push	235 (36.9)	401 (63.1)	.27†
Pull	252 (39.9)	379 (60.1)	
Drip	228 (35.8)	408 (64.2)	.065†
Bolus	259 (41.0)	372 (59.0)	
**Practice status**			
Faculty/In practice	212 (29.2)	514 (70.8)	<.001†
Trainee	275 (50.8)	266 (49.2)	
**Seen Patients Last 30 days at baseline**			
Yes	305 (35.8)	547 (64.2)	.007†
No	182 (43.9)	233 (56.1)	
**Paid CE Fee**			
Yes	133 (22.7)	454 (77.3)	<.001†
No	354 (52.1)	326 (47.9)	
**Herb Use**, baseline	5.2 ± 6.9	5.7 ± 5.9	.002 ∏
Self rank on **computer knowledge^$^**	4.53 ± 1.12	4.28 ± 1.16	<.001*
**Baseline Scores**			
Knowledge, % correct	64.0 ± 10.5	66.9 ± 10.5	<.001*
Confidence Scale Score	50.6 ± 18.4	53.6 ± 18.0	.005*
	N = 462	N = 738	
Communications	2.1 ± 1.8	2.2 ± 2.0	.75 ∏
Practices Scale Score	N = 305	N = 546	

* Determined by t test.

† Determined by Chi square test with continuity correction for 2 × 2 tables.

‡ Student completion rate less than each of the other four groups in pairwise comparisons, all P <.001; physicians/physician assistants less than nutritionists (P = .01) and nurses/nurse practitioners (P = .026); physician versus pharmacists P = .067.

∏ Determined by Mann Whitney U test.

^$ ^On this self-report scale, 1 = Novice and 7 = Trainer/Specialist.

**Table 3:** Outcome measures by randomized delivery strategy group.

**Characteristic**	**Total**	**Randomized Group**				**P Value**
		
		**Push + drip**	**Push + bolus**	**Pull + drip**	**Pull + bolus**	
**Knowledge questions (N)**	N = 780	N = 206	N = 195	N = 202	N = 177	
%Correct, Outcome	89.0 ± 9.5	88.3 ± 9.9	89.8 ± 8.2	89.4 ± 9.6	88.3 ± 10.1	.30*
Change in %Correct	22.0 ± 13.2#	21.9 ± 13.3	21.5 ± 12.0	23.5 ± 13.8	20.8 ± 13.6	.23*
**Confidence Scale Score (N)**	n = 735	n = 196	n = 186	n = 192	n = 161	
Outcome Score	64.3 ± 15.1	65.6 ± 13.8	63.8 ± 15.9	63.2 ± 15.1	64.4 ± 15.5	.42*
Change in Score	10.5 ± 22.8#	11.4 ± 22.8	9.6 ± 22.6	9.8 ± 24.5	11.1 ± 21.3	.89 †
**Communications Practices Scale (N)**	n = 440	n = 115	n = 115	n = 111	n = 99	
Outcome Score	2.0 ± 2.3	1.9 ± 2.2	2.4 ± 2.5	2.0 ± 2.2	1.9 ± 2.2	.27 †
Change in Score	0.33 ± 1.7‡	0.25 ± 1.7	0.30 ± 1.7	0.45 ± 2.0	0.30 ± 1.6	.36 †

This table includes only participants who completed scales at both baseline and outcome.

* Determined by one-way ANOVA.

† Determined by Kruskal Wallis test.

‡ P < .001 for change in use between baseline and outcomes (Wilcoxon signed rank test). This measure required the respondent to have seen patients in the past 30 days both at baseline and outcomes assessments.

# P < .001 for change between baseline and outcomes (paired samples t test).

^ N = 780 for these determinations. P < .001 for change in use between baseline and outcomes (Wilcoxon signed rank test)

**Table 4:** Factors Associated with Improvements in Knowledge, Confidence and Communication.

**Characteristic**	**Change in %Correct,** **Knowledge Questions**		**P Value**	**Change in Confidence** ** Scale Score**		**P Value**	**Change in Communications** ** Practices Scale Score**		**P Value**
	**N**	**(mean ± SD)**		**N**	**(mean ± SD)**		**N**	**(mean ± SD)**	

Overall change from baseline	768	22.0 ± 13.2		735	10.5 ± 22.8		440	.33 ± 1.74	
**Gender**									
Male	177	21.8 ± 14.2	.18*	164	11.8 ± 20.6	.72#	110	.26 ± 1.55	.52#
Female	591	22.0 ± 12.9		571	10.1 ± 23.5		330	.35 ± 1.81	
**Delivery Method**									
Push	394	21.7 ± 12.7	.58*	382	10.5 ± 22.6	.79#	230	.27 ± 1.69	.16#
Pull	374	22.2 ± 13.7		353	10.4 ± 23.1		320	.38 ± 1.81	
Drip	402	22.7 ± 13.5	.10*	388	10.6 ± 23.6	.65#	226	.35 ± 1.85	.74#
Bolus	366	21.2 ± 12.8		347	10.3 ± 22.0		214	.30 ± 1.62	
**Practice Status**									
Trainee	263	22.9 ± 15.1	.84*	248	15.7 ± 21.4	<.001#	112	.28 ± 1.55	.56#
Faculty/Practice	505	21.5 ± 12.1		487	7.8 ± 23.1		328	.34 ± 1.81	
**Seen Patients in last 30 days**									
No	231	24.9 ± 14.7	<.001*	216	14.6 ± 22.7	<.001#			
Yes	537	20.7 ± 12.3		519	8.8 ± 22.7		Required for this measure		
**Paid CE Fee**									
No	319	20.5 ± 15.0	.012*	307	7.4 ± 23.8	.006#	122	.43 ± 1.57	.31#
Yes	449	23.0 ± 11.6		428	12.7 ± 21.9		318	.28 ± 1.81	
**Age**									
≤30 years	198	24.4 ± 15.0	.005†	187	15.4 ± 22.2	.001^	83	.27 ± 1.57	.51^
31–40 years	121	19.6 ± 12.0		117	7.0 ± 24.0		75	.54 ± 1.60	
41–50 years	229	20.8 ± 12.0		220	7.4 ± 23.1		145	.20 ± 1.72	
>50 years	220	22.4 ± 13.0		211	11.3 ± 21.8		137	.38 ± 1.94	
**Professional Group**									
Nutrition/Dietician	110	23.4 ± 10.6	<.001†	111	2.9 ± 24.9	<.001^	60	.37 ± 1.84	.28#
Nurse/NP	205	23.3 ± 12.5		195	12.4 ± 23.8		126	.29 ± 2.06	
Physican/PA	231	18.6 ± 12.4		218	9.2 ± 21.5		176	.44 ± 1.59	
Pharmacist	43	20.0 ± 12.3		43	-3.4 ± 18.3		17	.56 ± 0.93	
Student	179	24.4 ± 15.6		168	18.5 ± 19.6		61	-.05 ± 1.50	
**No. Herbs and Supplement Used**			<.001†			.76^			.47^
0	111	24.4 ± 15.0		103	7.8 ± 26.5		55	.32 ± 1.46	
1–3	241	23.5 ± 12.8		232	11.1 ± 24.4		133	.32 ± 1.57	
4–8	238	21.8 ± 13.8		231	10.6 ± 23.1		146	.47 ± 1.82	
9+	178	18.7 ± 11.0		169	11.0 ± 17.3		106	.14 ± 1.98	
**Computer Expertise Self-Report Scale**			.003‡			.058‡			.50‡
1 (Novice)	16	29.5 ± 11.9		12	23.9 ± 16.8			.52 ± 1.48	
2	38	20.9 ± 12.9		39	7.7 ± 25.7			.28 ± 1.37	
3	124	24.3 ± 12.0		120	12.4 ± 22.2			.33 ± 1.87	
4	227	22.7 ± 12.7		214	11.6 ± 22.9			.43 ± 1.65	
5	271	20.0 ± 12.9		264	9.1 ± 23.1			.25 ± 1.92	
6	85	23.1 ± 15.9		81	9.4 ± 21.7			.26 ± 1.45	
7 (Expert)	7	11.7 ± 12.8		5	-1.0 ± 25.3			-.17 ± 1.04	

* T test in conjunction with Levene test for equal variances.

† One-way ANOVA. Pairwise analysis of subgroups not performed.

‡ Spearman's rho for correlation of ordinal with continuous variable (correlation coefficient = -.11 for knowledge change and -.07 for confidence change).

# Mann Whitney test.

^ Kruskal Wallis test. Pairwise analysis of subgroups not performed.

Notes: The Confidence Scale ranges from 19 to 95. A change in score of 19 is the equivalent of moving from one Likert scale category (Strongly Disagree, Disagree, Neutral, Agree, Strongly Agree) to an adjacent category for each of the 19 items in the scale. A change of 10 could represent similar change for 10 of 19 items, a change of two levels in 5 items, etc.

The Communications Practices Scale ranges from 0 to 10. A change of 1.0 would be the approximate equivalent of increasing the frequency of practices by 10% in each of the 9 percentage items in the scale. A change of 0.2 could represent an increase in frequency of 20% in a single item.
